# Evaluation of Potato Varieties for Yield, Quality, and Late Blight Resistance

**DOI:** 10.3390/life15091378

**Published:** 2025-09-01

**Authors:** Rita Asakaviciute, Avrelia Zelya, Tatiana Andriychuk, Almantas Razukas

**Affiliations:** 1 Lithuanian Research Centre for Agriculture and Forestry, Zalioji a. 2, Trakų Vokė, LT-02232 Vilnius, Lithuania; almantas.razukas@lammc.lt; 2 Ukrainian Science-Research Plant Quarantine Station Institute of Plant Protection National Academy of Agrarian Science, Chernivtsi District, Chernivtsi Region, 60321v Boyani, Ukraine; tatyjana58@gmail.com

**Keywords:** breeding, collection, *Phytophthora infestans*, potato, varieties

## Abstract

This study provides the first long-term, cross-border evaluation of Lithuanian potato (*Solanum tuberosum* L.) cultivars, integrating agronomic performance, tuber quality, and resistance to major pathogens across diverse environments. Field and controlled trials conducted in Lithuania and Ukraine from 2014 to 2024 revealed substantial genetic variability among 14 national cultivars, enabling their classification into five distinct maturity groups. Maincrop cultivars outperformed others in yield and starch accumulation, with ‘VB Meda’, ‘Goda’, and ‘VB Aista’ exhibiting a superior balance of productivity (up to 49 t ha^−1^), starch content (>19%), and moderate-to-high resistance to *Phytophthora infestans*. A broader genetic screening of 287 accessions—including varieties, breeding lines, and hybrids—demonstrated wide diversity in phenological development, disease resistance, and reproductive traits. Notably, Ro_1_ pathotype resistance was identified in 85 genotypes, predominantly with yellow-skinned tubers, while genotypic sterility in flowering and berry set was associated with both parental lineage and elevated temperatures. Although no complete immunity to *P. infestans* was detected, several genotypes displayed stable polygenic field resistance, suggesting the presence of horizontally inherited defense mechanisms effective under variable agroclimatic conditions. These results underscore the strategic breeding potential of Lithuanian potato germplasm for developing high-performing cultivars with enhanced resilience to late blight and nematodes and offer valuable insights for climate-adapted potato breeding in Northern and Eastern Europe.

## 1. Introduction

The potato (*Solanum tuberosum* L.) is a perennial plant belonging to the nightshade family (*Solanaceae*) [1]. Originating in the Andean region of South America, potatoes were introduced into European agriculture and diets in the sixteenth century [2]. Today, potatoes rank as the fourth most cultivated food crop globally, following wheat, rice, and maize [3]. Its global significance stems from its versatility: potatoes are consumed as food, used as animal feed, serve as seed tubers, and provide raw materials for both the food and non-food industries. The food sector processes potatoes into products such as crisps, chips, flakes, and canned goods, while the industrial sector uses them for starch, alcohol, and other derivatives [4].

In Lithuania, the Vokė Branch of the Institute of Agriculture under the Lithuanian Research Centre for Agriculture and Forestry (LAMMC) plays a central role in potato breeding and primary seed production. The institute also houses the national potato gene bank and maintains a diverse collection of more than 287 potato varieties and hybrids sourced globally. These genetic resources support breeding programs aimed at developing varieties with enhanced resistance to abiotic stress, diseases, and pests [5]. Over the years, these programs have successfully generated high-yielding and high-quality potato varieties through meristem-based propagation and rigorous selection methods [6].

Human and environmental factors significantly influence the variability and effectiveness of potato breeding. Addressing challenges such as quality improvement, disease resistance, and environmental sustainability requires strategic use of genetic diversity and innovative breeding approaches [7,8].

One of the most critical constraints in potato production is disease pressure, particularly from late blight caused by *Phytophthora infestans* (Mont.) de Bary. This disease remains one of the most economically devastating threats to potato crops worldwide, capable of reducing yields by 10–20 t ha^−1^ under adverse weather and disease conditions [9,10]. Late blight is especially problematic in regions with high relative humidity, cool nights, and warm summer days. The disease causes rapid foliage decay, reducing the photosynthetic surface and, thus, compromising tuber yield [11]. Despite extensive research and management efforts, the pathogen remains difficult to control due to its high genetic adaptability [12].

Resistance to *P. infestans* is a major focus in potato breeding. The genetic basis of late blight resistance is complex, involving major resistance (R) genes and numerous quantitative trait loci (QTLs) that, together, contribute to polygenic resistance [13]. *Solanum demissum* has been identified as a valuable source of effective resistance genes [14]. However, the durability of resistance is challenged by the pathogen’s evolving race structure. Narrow genetic diversity in the breeding material can result in widespread susceptibility among newly developed varieties once the pathogen overcomes the deployed resistance genes. For example, formerly resistant cultivars in Europe have succumbed to disease due to rapid changes in pathogen populations [10].

Consequently, sustainable potato production depends on the continuous development of varieties with broad-spectrum and durable resistance. Breeding strategies must begin with the collection and evaluation of diverse genetic material, followed by its integration into breeding pipelines. The evaluation of Lithuanian potato varieties for resistance to late blight is essential for identifying promising candidates for future cultivar development.

The hypothesis of this study: Lithuanian potato cultivars exhibit significant genetic variation in maturity, tuber quality, and resistance to Phytophthora infestans, and this variation can be effectively characterized through multi-year, multi-location trials. Furthermore, cultivars with a higher starch content and specific maturity groups will demonstrate greater resilience to late blight and nematode infection. Identifying these traits will enable targeted breeding of climate-resilient, high-yielding potato varieties adapted to Northern and Eastern European agroclimatic conditions.

The Lithuanian potato-breeding program has prioritized the development of early- and main-crop varieties with enhanced disease and pest resistance, particularly suitable for organic farming systems. The present study aimed to explore genetic diversity within the national potato collection, identify genotypes with valuable traits, and integrate them into breeding programs. Specifically, this study evaluated the resistance of selected Lithuanian potato varieties to *P. infestans* under variable climatic and pathogen conditions in both Lithuania and Ukraine. The conceptual novelty lies in linking polygenic field resistance tolerance with agronomic and phenotypic traits under real-world conditions, thus supporting the development of resilient cultivars suited to current and future agroclimatic challenges.

## 2. Materials and Methods

### 2.1. Study of Potato Genetic Resources Collection in Lithuania

This study was conducted on 287 potato cultivars and hybrids, representing genetic material collected from diverse global origins. Field trials were carried out from 2014 to 2024 at the Breeding Department of the Vokė Branch, the Institute of Agriculture, the Lithuanian Research Centre for Agriculture and Forestry.

The experimental site was established on sandy loam over carbonaceous fluvial–glacial gravel eluviated soil, classified as Haplic Luvisols (LVh) according to the FAO-UNESCO system. Trials were conducted in a crop rotation system on soddy podzolic sandy loam soil. The natural fertility of the soil was moderate, with a humus content of up to 2.0%, a pH_KCl ranging from 5.1 to 5.5, an available phosphorus (P_2_O_5_) content of 180–240 mg kg^−1^, and an available potassium (K_2_O) content of 150–190 mg kg^−1^.

Each cultivar was planted in separate 4.9 m^2^ field plots containing two rows with 20 tubers per plot. Planting was carried out manually when the soil temperature reached 7–10 °C. Fertilization was applied locally at a rate of N_90_P_90_K_90_ at planting. Field management included two harrowings before sprouting and two hillings during the growing season. Fungicides against *P. infestans* were applied once per season, and insecticides targeting Leptinotarsa decemlineata and aphids were used as needed. Harvesting was performed manually at the end of the vegetation period. Tubers were stored at +2 °C and 80–90% relative humidity throughout the winter.

The phenological and morphological characteristics of the plants were recorded using the BBCH scale, including over 50 traits. Key traits evaluated included sprouting onset, plant vigor, flowering period, flower color, stem number, disease symptoms, foliage senescence, and the end of the vegetation period. Cultivars were categorized into five maturity groups: very early, early, main crop, late, and very late. Foliar resistance to bacterial, fungal, and viral pathogens was visually assessed during flowering. Yield and quality traits were evaluated following Razukas et al. [5] and included total yield, marketable yield, tuber number, tuber shape, eye depth, skin and flesh color, dry matter content, and cooking and processing characteristics.

### 2.2. Screening for Resistance to Potato Late Blight in Lithuania

Gene bank potato varieties and seedlings during the vegetation period were tested for their resistance to diseases. The trials were set up in conformity with the local agricultural potato growing practices. Disease severity was measured by the scale approved and recommended by OEPP/EPPO [9]. One hundred plants from every plot were tested. The potato vine infection level was scored according to the attack: few plants with lesions—1–2 lesions in a 10 m radius; 0.5—1–5 spots per plant; 1—5–10 spots per plant; 5—about 50 spots per plant, or up to 1 in 10 leaflet lesions; 10—about 10% of the leaf area is destroyed, up to 4 in 10 leaves are destroyed, and nearly every leaflet has lesions, but the plants still look normal; 25—about 25% of the leaf area is destroyed, nearly every leaflet has lesions, the plants remain normal, and the field still looks green; 50—about 50% of the leaf area is destroyed, every plant has lesions, and the field is still green but with brown spots; 75—about 75% of the leaf area is destroyed, and the field color is between green and brown; 95—only a few leaves are left, but the stems are still green; and 100—all leaves are dead or dying. Late blight data obtained from the trial field were scored by percentage. Late blight spread was assessed, and the first early potato cultivar, ’VB Venta’, was used as a control.

The following formulas were used to calculate disease spread and intensity: disease spread (P) = (n × 100)/N, where P is the spread of disease (%), n is the number of infected plants or tubers, and N is the total number of assessed plants or tubers; disease intensity (R) = ∑(x)/N, where R is the disease intensity (%), ∑(x) is the sum of severity ratings multiplied by the number of plants in each severity group, and N is the total number of assessed plants or tubers.

### 2.3. Screening for Resistance to Potato Late Blight in Ukraine

Resistance to late blight was also assessed under natural infection conditions in Ukraine, following the methodologies of Bychenkova [15] and Golyachuk and Kosylovych [16]. Trials were carried out in 50 m^2^ plots, with 100 plants per cultivar (25 plants in four replications). The intensity of late blight disease was recorded on a 9-point scale [17,18].

Field studies were conducted on a natural infectious background, where the most common race of late blight in Ukraine was 1.2.3.4.5.6 + 0.7.8.9.10.11 xyz. The first registration was carried out when single symptoms of the disease appeared on the tested material for each bush, and the following ones, every 7 days, until the potato tops died. The degree of infection was scored on a 9-point scale: 9–8 indicated very high resistance (symptoms of infection were absent); 7–6 indicated relatively high resistance (with the infected tissue ranging from 10% to 25% of the surface and cut from tubers); 5–4 indicated moderate resistance (infected from 25% to 50%); 3–2 indicated low resistance (infected from 50% to 75%); and 1 indicated very low resistance (infected by more than 75%) [18].

The average resistance score was determined by summing the scores for each plant and dividing the sum by the number of records. Disease spread and intensity were calculated using the same formulas as in the Lithuanian trials [19]: P = (n × 100)/N and R = ∑(x)/N.

### 2.4. Statistical Data Analysis

Statistical analysis of the experimental data was performed using the StatView software package, version X.X (SAS Institute Inc., Cary, NC, USA). The results were evaluated using analysis of variance (ANOVA), and significant differences between means were determined using the least significant difference (LSD_0.05_) method [20].

## 3. Results and Discussion

### 3.1. Results of Survey of Potato Genetic Resources Collection in Lithuania

One of the primary agronomic traits of potato cultivars is the duration of the growing season. Field studies conducted in southeastern Lithuania have enabled the efficient utilization of potato varieties according to the local photoperiod and climatic conditions. Day length exerts a direct influence on both the duration of vegetative growth and tuber yield. Potato varieties of South American origin are adapted to short-day conditions (13–14 h) and are classified accordingly [4].

The Lithuanian potato genetic resources collection is divided into five maturity groups based on the length of the growing period (from sprouting to leaf senescence): very early maturing (52–58 days), comprising 49 varieties; early maturing (59–68 days), comprising 62 varieties; medium maturing (69–75 days), comprising 42 varieties; late maturing (76–85 days), comprising 20 varieties; and very late maturing (86–101 days), comprising 27 varieties.

A key consideration in potato hybrid breeding is the ability to flower and produce berries. Empirical evidence shows that modern varieties, which are more genetically distant from their progenitors, often exhibit reduced or absent berry formation. Over the past decade, 120 varieties in the gene bank have shown regular flowering, but only 67 varieties and hybrids have produced fully mature seeds under field conditions without artificial intervention. The primary limitation to berry and seed formation is sterility. In some cases, sterility results from the failure of flower development, with flowers reduced to 0.5–1 cm in size. In other cases, phenotypic stamen sterility was observed, often associated with adverse environmental conditions, such as high temperatures (25–30 °C) during the flowering phase. However, this sterility can be reversed under cool and moist conditions, where improved flowering and seed set are observed. Varieties with natural stamen sterility are valuable as maternal parents in breeding programs. Crosses between sterile and fertile lines typically segregate into sterile and fertile progeny. For fertile genotypes that produce berries, classical crossing methods and laboratory techniques—such as protoplast fusion—can be used to transfer desirable traits.

The Lithuanian potato gene bank is dominated by varieties with light yellow tuber skin and flesh coloration. In total, 135 varieties exhibit light yellow skin, and 122 varieties also display light yellow flesh. These traits are frequently associated with resistance to the Ro1 pathotype of the potato cyst nematode. Morphological features such as tuber shape, uniformity, and eye depth are critical selection criteria in breeding. Hybrids with poor shape or uniformity are typically excluded from breeding lines. A deterioration in tuber shape and uniformity has been observed with increasing age of the breeding population. Additionally, older varieties tend to show heightened susceptibility to viral infections and more pronounced deviations from cultivar standards. This phenomenon reflects the effect of entropy on long-term cultivated populations.

Maincrop varieties are the most productive under Lithuanian growing conditions, although they lack donors with resistance to late blight. Therefore, early- and late-maturing groups serve as sources of genetic material in the development of improved maincrop cultivars. Special techniques are applied to synchronize flowering time for successful hybridization. The following cultivars have been developed using material from the Lithuanian gene bank: ‘VB Meda’ (‘Matilda’ × N 3093), ‘VB Aista’ (N 263 × N 476-9), ‘Goda’ (‘Ausonia’ × ‘Franci’), ‘VB Liepa’ (N 34/36 × ‘Pirmūnės’), ‘Mėta’ (‘Sagitta’ × ‘Comtesa’), ‘Mirta’ (‘Fryla’ × No 17/6), ‘Nida’ (‘Amaryl’ × (‘Sagitta’ × ‘Olev’)), ‘Pirmūnės’ (‘Pepo’ × VIR), ‘VB Rasa’ (‘Cardinal’ × ‘Viola’), ‘Vokė’ (‘Majestic’ × No 323), ‘Vilnia’ (‘Sagitta’ × ‘Neringa’), ‘Vaiva’ (‘Hanibal’ × ‘Anosta’), ‘VB Venta’ (‘Priekulu visagrie’ × ‘Pirmūnės’), and ‘Vilija’ (‘Voltman’ × ‘Pepo’).

Considerable attention is devoted to resistance against diseases and pests. All varieties and hybrids in the gene bank are immune to wart disease. In total, 85 varieties are resistant to the Ro1 nematode pathotype. When both parent varieties are resistant, their progeny are typically immune. However, if only one parent is resistant, the offspring may segregate for resistance. Virus infections remain a major challenge in potato cultivation, contributing to reductions in yield and quality. No variety or hybrid has complete resistance to all viruses, and all newly developed varieties become infected during vegetative growth. Consequently, the use of virus-tolerant genotypes in breeding programs is essential. Susceptible varieties rapidly degrade under viral pressure, becoming more vulnerable to bacterial and fungal diseases. In contrast, tolerant varieties exhibit only mild symptoms under optimal conditions. A notable example of virus tolerance is the variety ‘Dietskosielskij’, which is actively used in Lithuanian breeding efforts. Tolerance to viral infections in new varieties is achieved using classical selection methods.

**Figure 1:**
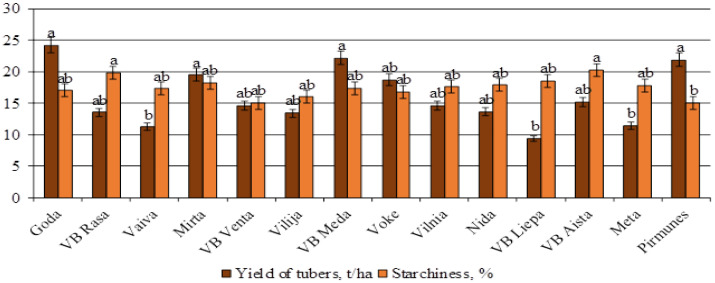
Economic parameters characteristic of various Lithuanian potato varieties in Lithuania. Each value is the mean of three replicates ± standard error; LSD_0.05_-2.141. (Voke branch, 2014–2024).

Data on the agronomic traits and tuber quality of selected Lithuanian varieties are presented in Figure 1. All varieties exhibited a high seed potato yield, depending on their genetic potential. Starch content varied significantly among genotypes, with the starch-processing variety ‘VB Aista’ demonstrating the highest starch yield at 20.3 ± 0.97%. Early-maturing varieties accumulated starch up to 18%. The number of tubers per plant was also variety-dependent, with ‘Goda’ producing the highest tuber number. Early and maincrop varieties tended to produce larger tubers than late-maturing ones. The highest tuber weights were recorded for ‘VB Venta’, ‘Nida’, and ‘Goda’. Susceptibility to disease was closely related to genetic origin, confirming the importance of parental selection in resistance breeding.

### 3.2. Evaluation of Late Blight Resistance in Lithuanian Potato Varieties

Potato yields are known to decrease by 15–50% annually due to late blight, with losses reaching up to 80% in years when epiphytotic outbreaks occur [21]. The severity of damage caused by late blight (*P. infestans*) is influenced by numerous factors, including the location of the crop, prevailing meteorological conditions, agronomic practices, disease onset, varietal resistance, and the timeliness and effectiveness of protective measures [11].

**Figure 2:**
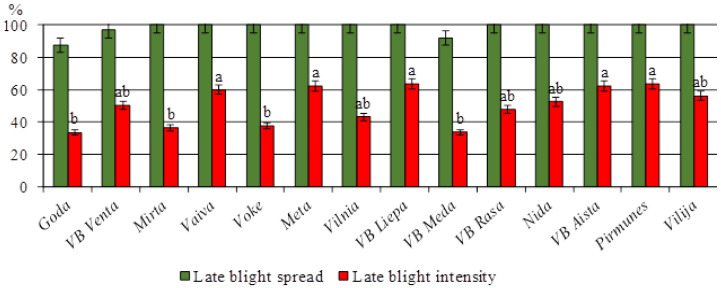
Resistance of foliage of Lithuanian potato varieties under natural infection pressure in Lithuania. Each value is the mean of three replicates ± standard error, LSD_0.05_-3.287 (Voke branch, 2014–2024).

Surveys conducted from 2014 to 2024 at the Vokė branch of LAMMC focused on the resistance of potato varieties to *P. infestans*. This research is a cornerstone in the development of new potato varieties with enhanced resistance or immunity to late blight. Comprehensive evaluation of breeding materials revealed that among the 14 Lithuanian varieties, ‘Goda’, ‘VB Meda’, ‘Mirta’, and ‘Vokė’ demonstrated the highest levels of resistance (Figure 2).

Late blight remains the most economically devastating potato disease in Lithuania. The pathogen reduces photosynthetic areas by destroying foliage during tuber formation, leading to substantial yield losses and the development of various storage roots. Despite extensive testing over a decade, no potato variety has exhibited complete immunity to *P. infestans*. In general, varieties in the first early, second early, and maincrop maturity groups demonstrated low levels of resistance.

In terms of long-term disease management, resistance breeding strategies based on single major resistance genes are proving less effective due to evolving pathogen virulence. As a result, breeders are increasingly shifting toward the development of varieties with polygenic (field) resistance, which relies on the combined effect of multiple minor genes. These genes do not confer complete immunity but significantly slow pathogen progression, allowing the plant to better tolerate infection [12]. This approach supports a more durable form of resistance.

To ensure reliable assessment of varietal resistance, future potato cultivars and hybrids should be evaluated both under field conditions with natural infections and laboratory conditions with controlled inoculations [5,13]. Such comprehensive testing allows for the identification of differences in resistance based on genetic background, maturity group, and year-to-year environmental variability.

Key meteorological variables that influence late blight progression include precipitation levels, average daytime temperatures, and humidity, especially from the onset of disease development until the peak of damage. The present study demonstrates that the intensity and timing of late blight infection are influenced by both the maturity group and the biological–genetic characteristics of the potato variety.

Based on multi-year field observations, Lithuanian potato cultivars were classified into three resistance groups: highly susceptible (B1), including ‘VB Aista’, ‘Pirmūnės’, ‘Mėta’, ‘VB Liepa’, and ‘Vaiva’; moderately susceptible (B2), including ‘VB Venta’, ‘Vilnia’, ‘VB Rasa’, ‘Nida’, and ‘Vilija’; and moderately resistant (B3), including ‘Goda’, ‘Vokė’, ‘Mirta’, and ‘VB Meda’.

Notably, the late-maturing varieties ‘Vilnia’ and ‘Vokė’ showed the greatest resistance to late blight across all testing years. In contrast, early-maturing varieties were more heavily affected, with widespread infection of leaves, stems, apices, and tubers. The findings confirm that early maturity is often associated with increased vulnerability to *P. infestans* due to prolonged periods of exposure during favorable infection conditions.

### 3.3. Evaluation of Lithuanian Potato Varieties for Late Blight Resistance in Ukraine

**Figure 3:**
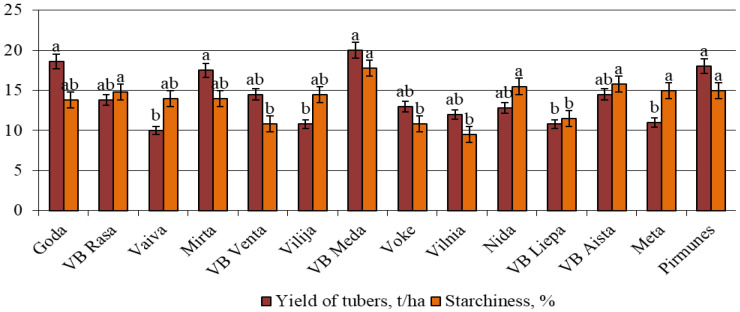
Economic parameters characteristic of various Lithuanian potato varieties in Ukraine. Each value is the mean of three replicates ± standard error; LSD_0.05_-1.947 (UkrSRPQS IPP, 2019–2024).

The quality performance of the Lithuanian potato varieties tested in Ukraine is presented in Figure 3. The data illustrate the variations in both tuber yield and starch content across 14 Lithuanian potato varieties: the highest tuber yields (≥18 tha^−1^) were observed in ‘VB Meda’, ‘VB Aista’, and ‘Goda’, with statistically significant superiority (denoted by “a”). The highest starch content (≥16%) was also recorded in ‘VB Aista’, VB Meda’, and ‘Goda’, highlighting their dual potential for both productivity and processing quality. ‘Pirmūnės’, ‘Vilija’, and ‘VB Liepa’ exhibited lower yield and starch content, suggesting more limited agronomic or industrial use under the Ukrainian test conditions.

Several varieties (e.g., ‘VB Venta’, ‘Vilnia’, and ‘Vokė’) fell into intermediate groups for both parameters, showing balanced but not peak performance.

These results underscore the suitability of ‘VB Meda’, ‘Goda’, and ‘VB Aista’ as candidates for cultivation in Ukraine, given their combination of moderate-to-high resistance to late blight and superior agronomic performance.

**Figure 4:**
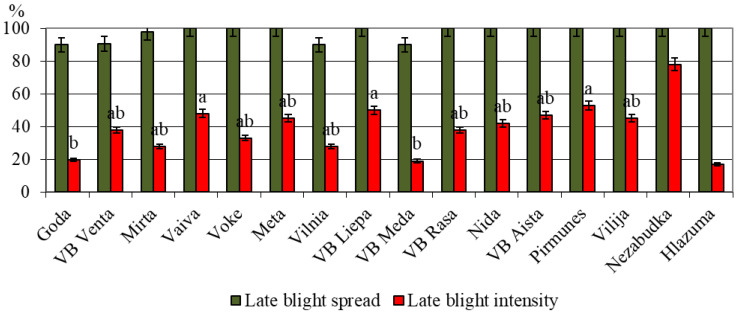
Resistance of foliage of Lithuanian potato varieties under natural infection pressure in Ukraine. Each value is the mean of three replicates ± standard error; positive control: ‘Nezabudka’; negative control: ‘Hlazurna’; LSD_0.05_-2.878 (UkrSRPQS IPP NAAS, 2019–2024).

The present study includes results from trials conducted at the Ukrainian Research Station of Plant Quarantine under the Institute of Plant Protection, the National Academy of Sciences of Ukraine, which aimed to evaluate the resistance of Lithuanian-bred potato varieties to *P. infestans*. The trials were performed over the period of 2019–2024 under both controlled and field conditions (Figure 4).

The analysis revealed considerable variation among the tested varieties in terms of both disease spread and intensity. Although the late blight spread was relatively high and similar across all varieties (ranging from 85% to 95%), notable differences were observed in late blight intensity, indicating differences in varietal susceptibility.

Among the tested varieties, ‘VB Meda’, ‘Goda’, and ‘Mirta’ demonstrated the lowest intensity of late blight symptoms (approximately 30–35%), indicating a higher level of resistance. These varieties form a statistically distinct group (denoted by the letters ‘a’ and ‘ab’) and can be considered as resistant genotypes. Moderate resistance was also observed in ‘VB Venta’, ‘VB Rasa’, ‘VB Aista’, and ‘Vokė’, with intensity levels remaining below 45%. The variety ‘Pirmūnės’ showed the highest disease incidence among Lithuanian lines, with 53% leaf and stem damage.

Two control varieties were used for benchmarking resistance: The negative control, ‘Glazurnaya’, recorded only 17% damage, indicating high resistance. The positive control, ‘Nezabudka’, exhibited 76% infection of aerial parts and rapid tuber involvement under both laboratory and field conditions.

These findings suggest that while late blight is capable of spreading rapidly under conducive conditions, certain Lithuanian-bred varieties exhibit enhanced genetic resistance, particularly in limiting the severity of infection. The identification of these resistant cultivars provides valuable information for future breeding programs aimed at improving late blight tolerance.

## 4. Conclusions

This study highlights the diversity and agronomic potential of Lithuanian potato cultivars, with particular emphasis on their maturity groups, tuber quality traits, and resistance to late blight (Phytophthora infestans). Field trials conducted in Lithuania and Ukraine from 2014 to 2024 confirmed that maincrop varieties are generally the most productive under Lithuanian growing conditions. Among the tested cultivars, ‘VB Meda’, ‘Goda’, and ‘VB Aista’ demonstrated a superior combination of high tuber yield, elevated starch content, and moderate-to-high resistance to late blight, making them suitable candidates for both national and regional cultivation and breeding programs. It is recommended that these varieties be incorporated into agricultural production in Lithuania and Ukraine.

The evaluation of flowering and berry production under natural conditions revealed significant variation among genotypes, with sterility—whether structural or environmentally induced—posing a constraint for hybridization. Nonetheless, genotypes with natural male sterility offer value as maternal lines in breeding strategies. The high prevalence of light-yellow skin and flesh coloration among Lithuanian varieties, often associated with Ro1 nematode resistance, further underscores the importance of combining phenotypic and genetic markers in selection.

Late blight remains the most economically damaging disease affecting potatoes in Lithuania. Although none of the studied cultivars exhibited full immunity, several demonstrated polygenic field resistance, which slows disease progression and supports more sustainable crop protection. Resistance was found to vary not only by genetic background but also by maturity group and environmental conditions, with late-maturing varieties showing generally higher tolerance.

These findings confirm the value of the Lithuanian potato gene pool in addressing current agronomic challenges, particularly those related to disease resistance, yield stability, and quality. Continued multi-environmental testing and integration of resistant genetic resources into breeding programs are essential for developing cultivars adapted to changing climatic and phytopathological conditions.

## Data Availability

The raw data supporting the conclusions of this article will be made available by the authors on request by email.

## References

[1] Struik P.C., Vreugdenhil D., Bradshaw J., Gebhardt C., Govers F., MacKerron D.L., Taylor M.A. (2007). Above-ground and below-ground plant development. Potato Biology and Biotechnology—Advances and Perspectives.

[2] Rocha A.M.C.N., Coulon E.C., Morais A.M.M.B. (2003). Effects of vacuum packaging on the physical quality of minimally processed potatoes. Food Serv. Technol..

[3] Vreugdenhil D., Bradshaw J., Gebhardt C., Govers F., Taylor M.A., MacKerron D.K., Ross H.A. (2007). Potato Biology and Biotechnology—Advances and Perspectives.

[4] Ríos D., Devaux A., de Galarreta J.I.R. (2024). Ancient Potato Varieties of the Canary Islands: Their History, Diversity and Origin of the Potato in Europe. Potato Res..

[5] Razukas A., Jundulas J., Asakaviciute R. (2008). Potato cultivars susceptibility to potato late blight (*Phytophthora infestans*). Appl. Ecol. Environ. Res..

[6] Asakaviciute R., Razukas A., Jundulas J. (2009). Susceptibility of new potato varieties to the potato late blight oomycete *Phytophthota infestans* (mont.) De Bary in Lithuania. Agrociencia.

[7] Brazinskiene V., Asakaviciute R., Miezeliene A., Alencikiene G., Ivanauskas L., Jakstas V., Viskelis P., Razukas A. (2014). Effect of farming systems on the yield, quality parameters and sensory properties of conventionally and organically grown potato (*Solanum tuberosum* L.) tubers. Food Chem..

[8] Asakaviciute R., Kacergius A., Razukas A. (2016). Breeding aspects of potato in Lithuania. Proc. Appl. Bot. Genet. Breed..

[9] Schepers H.T.A.M. (2000). The development and control of *Phytophthora infestans* in Europe in 1999. PAV-Spec. Rep..

[10] Hansen J.G., Koppel M., Valskyte A., Turka I., Kapsa J. (2005). Evaluation of foliar resistance in potato to *Phytophthora infestans* based on an international field trial network. Plant Pathol..

[11] Rymuza K., Radzka E., Lenartowicz T. (2015). The effect of weather conditions onearly potato yields in east-central Poland. Commun. Biometry Crop Sci..

[12] Kroon L.P.N.M., Henk B., de Cock A.W.M., Govers F. (2011). The Phytophthora Genus Anno. Phytopathology.

[13] Asakaviciute R., Zelya A., Kacergius A., Andriychuk T., Zelya G., Skoreyko A., Razukas A. (2025). Assessment of potato varieties of Lithuanian breeding resistance potato wart causative agents and late blight. Sci. Rep..

[14] Ballvora A., Ercolano M.R., Weib J., Meksem K., Bormann C.A., Oberhagemann P., Salamini F., Gebhardt C. (2012). The R1 gene for potato resistance to late blight (*Phytophthora infestans*) belongs to the leucine zipper/NBS/LRR class of plant resistance genes. Plant J..

[15] Bychenkova A.A. Types of field resistance of potatoes to late blight. Proceedings of the 5th All-Union Conference on Plant Immunity.

[16] Golyachuk Y.S., Kosylovych G.O. (2018). Genetic structure of populations of the pathogen of late blight of potatoes in the conditions of the Western Forest-Steppe of Ukraine. Bull. Lviv. Natl. Agrar. Univ. Ser. Agron..

[17] Kutsenko V.S., Osypchuk A.A., Podhaetskyi A.A. (2002). Methodical Recommendations for Conducting Research with Potatoes.

[18] Cherednychenko L., Furdyga M., Sobran V., Suchkova V. (2021). Assessment of resistance against late bligth of potato on leaves of newly created and original selection material of potato. Bull. Agric. Sci..

[19] Melnyk S.I. (2016). Methodology for Conducting Phytopathological Studies During Artificial Infection of Plants.

[20] Tarakanovas P. (2002). Data transformation of biological experiments using a computer program ANOVA. Zemdir-Byste-Agric..

[21] Asakaviciute R., Kacergius A., Razukas A. (2017). New Lithuanian potato varieties and their resistance to *Phytophthora infestans* (Mont.) de Bary. Bulg. J. Agric. Sci..

